# IL-32 is induced by activation of toll-like receptors in multiple myeloma cells

**DOI:** 10.3389/fimmu.2023.1107844

**Published:** 2023-02-16

**Authors:** Kristin Roseth Aass, Synne Stokke Tryggestad, Robin Mjelle, Martin H. Kastnes, Tonje Marie Vikene Nedal, Kristine Misund, Therese Standal

**Affiliations:** ^1^ Centre of Molecular Inflammation Research, Department of Clinical and Molecular Medicine, Norwegian University of Science and Technology, Trondheim, Norway; ^2^ Bioinformatics Core Facility - BioCore, Norwegian University of Science and Technology, Trondheim, Norway; ^3^ Department of Pathology, St. Olavs University Hospital, Trondheim, Norway; ^4^ Department of Hematology, St. Olavs University Hospital, Trondheim, Norway

**Keywords:** multiple myeloma, TLR, inflammation, IL-32, NFκB, cancer, cytokine

## Abstract

Multiple myeloma (MM) is a hematological cancer characterized by accumulation of malignant plasma cells in the bone marrow. The patients are immune suppressed and suffer from recurrent and chronic infections. Interleukin-32 is a non-conventional, pro-inflammatory cytokine expressed in a subgroup of MM patients with a poor prognosis. IL-32 has also been shown to promote proliferation and survival of the cancer cells. Here we show that activation of toll-like receptors (TLRs) promotes expression of IL-32 in MM cells through NFκB activation. In patient-derived primary MM cells, IL-32 expression is positively associated with expression of TLRs. Furthermore, we found that several TLR genes are upregulated from diagnosis to relapse in individual patients, predominantly TLRs sensing bacterial components. Interestingly, upregulation of these TLRs coincides with an increase in IL-32. Taken together, these results support a role for IL-32 in microbial sensing in MM cells and suggest that infections can induce expression of this pro-tumorigenic cytokine in MM patients.

## Introduction

Multiple myeloma (MM) is a hematological cancer characterized by accumulation of malignant plasma cells in the bone marrow. Clinical manifestations include high levels of monoclonal antibodies in serum and urine, anemia, multiple organ failure, immune suppression and bone disease (1). IL-32 is a pleiotropic cytokine with classical pro-inflammatory functions as well as more unconventional roles in cancer, autoimmune diseases, and infections (2–4). It is shown to be an important growth factor and metabolic regulator of MM cells (5). Furthermore, IL-32 is secreted by MM cells in exosomes and alters the tumor microenvironment by promoting osteoclastogenesis and bone degradation (6) as well as immune suppression (7, 8). High expression of IL-32 at diagnosis is observed in 10-15% of patients and these individuals have inferior survival compared to non-expressors (5, 6). Moreover, IL-32 expression is upregulated upon relapse in a fraction of patients (5).

IL-32 plays an important role in the host defense against a range of infectious agents (4) and IL-32 expression can be induced by toll-like receptor (TLR) activation in monocytes/macrophages, dendritic cells, and epithelial cells (9–13). TLRs recognize pathogen associated molecular patterns (PAMPs) and may also recognize and respond to danger associated molecular patterns (DAMPs) released during cell damage and cell death (14, 15). MM patients suffer from chronic and recurrent fungi-, bacteria- and virus- infections (16–18). In addition, due to cancer-induced inflammation and cancer treatment there is extensive cell death in the MM bone marrow microenvironment, which may lead to the release of DAMPS. Thus, MM cells in the bone marrow are likely to be exposed to TLR agonists derived from either microbes or cellular stress.

We have previously shown that IL-32 is induced in response to hypoxia (6). Whether IL-32 expression is induced in response to TLR signaling in MM cells is not known. Thus, to determine if infections or cell death may induce cancer cell expression of IL-32 we here examined if IL-32 is induced by TLR agonists in MM cell lines and if expression of IL-32 correlates with TLR expression in primary MM cells.

## Methods

### Cells and culture conditions

MM cell lines ANBL-6, INA-6 and JJN-3 were kind gifts from Dr. Diane Jelinek (Mayo Clinic, Rochester, MN), Dr. Martin Gramatzki (University of Erlangen-Nuremberg, Erlangen, Germany), and Dr. Jennifer Ball (University of Birmingham, UK), respectively. RPMI-8226 and U266 were obtained from American Type Culture Collection (ATCC, Rockville, MD, USA), while IH-1 and OH-2 were established in our laboratory (19, 20). RPMI-8226, U266 and JJN-3 were cultured in RPMI-1640 (RPMI) medium with 20, 15 and 10% heat inactivated fetal calf serum (FCS), respectively. ANBL-6 and INA-6 cells were cultured in RPMI with 10% FCS and the addition of 1 ng/ml recombinant human (rh) interleukin (IL)-6. OH-2 and IH-1 were cultured in RPMI containing 10% heat inactivated human serum (HS), and KJON-1 with 5% HS, both supplemented with 1 ng/mL rhIL-6. Cell lines were cultured at 37°C in a humidified atmosphere containing 5% CO_2_. RPMI-8226 TLR9 and TLR4 KO cells were generated using CRISPR/Cas9 technology followed by single cell cloning and maintained in the same culture conditions as the original RPMI cells. For LPS stimulation experiments with peripheral blood mononuclear cells (PBMCs) from healthy donors, PBMCs were isolated from fresh buffycoats by Histopaque -1077 gradient and kept in 10% RPMI medium overnight before stimulation-experiments. Freshly isolated primary myeloma cells were obtained from the local hospital biobank (Biobank1). CD138+ cells were isolated by RoboSep automated cell separator using Human CD138 Positive Selection Kit (StemCell Technologies, Grenoble, France) and later the same day, CD138+ cells were seeded in RPMI-1640 with 2% HS and 1 ng/mL rhIL-6 and stimulated with TLR ligands for 4 hours. When few primary myeloma cells were donated, the cells were stimulated with a cocktail containing all the ligands. Concentrations of ligands are described below. All patient samples were donated after informed consent, and the study was approved by the regional ethics committee (REK# 2011/2029 and REK# 247909). The study was performed in accordance with the Helsinki declaration.

### TLR9 and TLR4 CRISPR/Cas9 KO cell lines

RPMI-8226 cells were electroporated with TLR9 (#sc-400600) and TLR4 (sc-400068-KO-2) CRISPR/Cas9 KO and control (#sc-418922) plasmids from Santa Cruz Biotechnology, all containing green fluorescent protein (GFP) for selection. Cells were transfected using the Nucleofector™ II Device (Lonza) with buffer V (Amaxa Nucleofector Kit V, Lonza) and program G-015. Cells were then sorted for GFP positivity on a FACSAria Fusion flow cytometer (BD Biosciences) and single cell cloned. Clones were screened for TLR expression by immunoblotting (TLR9 KO and WT) or qPCR (TLR4 KO and WT).

### Ligands, inhibitors, and antibodies

For stimulation of cell lines and primary cells the following ligands were used: TLR2/1: Pam3Cys (EMC microcollections, Tübingen/Germany), TLR2/6: FSL-1 (EMC microcollections), TLR3: Poly (I:C) HMW (Invivogen, San Diego, CA, USA), TLR4: Ultrapure LPS (E.coli 0111:B4, Invivogen) TLR5: Flagellin (Invivogen), TLR7 and 8: R-848 (Invivogen), TLR9: CpG 2006 (TIBMolBiol, Berlin Germany). Based on titrations of ligands (**Supplemental Figures 2A, B, C**) the following concentrations were used: Pam3Cys: 1 µg/mL, FSL-1: 1 µg/mL, Poly(I:C): 10 µg/mL, LPS: 0.1 µg/mL, Flagellin: 1 µg/mL, R-848: 1 µg/mL, CpG: 1 µM.

For western blotting the following antibodies were used: anti- IL-32 (#AF3040, R&D Systems Minneapolis, MN, USA), anti β-actin (#4967), anti-TLR9 (#13674), anti- p-IκB(#2859), anti-IκB (#4812) all from Cell Signaling Technology (Danvers, MA, USA).

For inhibition of the NFκB pathway the IKK inhibitor VII (CAS 873225-46-8, Calbiochem, San Diego, CA, USA) and the TAK1 inhibitor NG25 (MedChemExpress, Monmouth Junction, NJ, USA) were used. The concentration used for NG25 was 2 μM and was based on previous titrations in MM cell lines (unpublished data, Starheim et al.). For IKK VII we used 10 μM, based on titrations as shown in (**Supplemental Figure 2F**). We pre-incubated the cells with inhibitors for 30 minutes before adding LPS for further 4 hours (time-point titration as shown in **Supplemental Figure 2G**).

### Sequencing of primary MM cells

Own dataset: CD138+ MM cells were obtained from Norwegian Myeloma Biobank (Biobank1, St. Olavs University Hospital HR, Trondheim, Norway) and isolated as previously described (6). Next, RNA was isolated from purified MM cells using miRVana total RNA isolation (ThermoFisher, #AM1560). RNA-seq was performed using the TruSeq Stranded mRNA Library Prep Kit (Illumina # RS-122-2101) according to the manufacturer’s protocol using 400 ng input RNA. Illumina (#20020595, San Diego, CA, USA) followed by 75 bp single read sequencing on the Illumina Hiseq 4000 next machine. The depth of sequencing was 18 million reads per sample. The study was approved by the regional ethics committee (REK # 247909).

### RNA sequencing data analyses

RNA sequencing data (MMRF_CoMMpass_IA13a_E74GTF_Salmon_Gene_Counts) and clinical data were downloaded from the Multiple Myeloma Research Foundation CoMMpass IA13 release (https://research.themmrf.org/). RNA sequencing data from CD138^+^ cells were available for 795 baseline samples from patients with MM. For TLR gene expression analysis, patient samples taken at diagnosis were divided into high and low IL-32 expression based on the same percentiles (upper 10^th^ and lower 90^th^) as used in our previous study on IL-32 in myeloma (5). We used the upper 10th percentile (n = 54; counts per million (cpm, log2)> 1.52) and lower 90th percentile (n = 741, (cpm,log2)≤ 1.52) and significance was analyzed by one-tailed Wilcoxon signed-rank test in R. Pearson correlation analysis was performed using *stat_cor* function in R.

RNA-sequenced CD138^+^ cells from longitudinal samples were available for 47 samples in IA13. We analyzed IL-32 expression at diagnosis and first relapse timepoint and divided these into two equally large groups based on fold change (FC) upregulation of IL-32 from diagnosis to relapse. Patients with log2 FC≥2.25 (n=23) and log2 FC<2.25 (n=24) were separated into two groups. Fold change differences in TLR expression between diagnosis (baseline) and relapse were calculated for these two groups.

For own RNA sequencing data (the Biobank1 dataset), high IL-32 expressing patients were defined as those with IL-32 expression (cpm,log2) >1.57, and low/non-expressing patients were defined as those with IL-32 expression (cpm,log2) ≤ 1.57), which represents the upper 30% percentile (n=26) and lower 70% percentile (n=61), respectively, This is approximately the same cpm cutoff as used when analyzing the CoMMpass IA13 dataset. Differences in TLR gene expression were assessed using one-tailed Wilcoxon signed-rank test in R.

All analyses were run using R version 3.6.2 (2019-12-12). Used packages with version number includes: packageVersion(“biomaRt”) ‘2.41.4’; packageVersion(“edgeR”) ‘3.26.8’; packageVersion(“ggplot2”) ‘3.2.1’. packageVersion(“ggpubr”) ‘0.4.0’.

### Real-time quantitative PCR

Total RNA was isolated using RNeasy kit (Qiagen, Hilden, Germany). Complementary DNA (cDNA) was synthesized from total RNA using High-Capacity RNA‐to‐cDNA kit (Applied Biosystems, Carlsbad, CA, USA). PCR was performed using StepOne Real‐Time PCR System and Taqman Gene Expression Assays (Applied Biosystems) using standard settings (2′ 50°C, 10 ′ 95°C, 40 cycles at 95°C for 15 sec, 1′ 60°C). Relative gene expression was analyzed by the comparative Ct method, and genes with Ct values >32 were regarded as detected. Probes were as follows: human IL-32 (Hs00992441_m1), TLR1 (Hs00413978_m1), TLR2 (Hs01872448_s1), TLR3 (Hs01551078_m1), TLR4 (Hs00152939_m1), TLR5 (Hs01019558_m1), TLR6 (Hs01039989_s1), TLR7 (Hs00152971_m1), TLR8 (Hs00152972_m1), TLR9 (Hs00152973_m1) and housekeeping gene TATA-binding protein (*TBP*; Hs00427620_m1) or *β*-actin (Hs0160665_g1).

### Immunoblotting

Cells were lysed in lysis buffer (50 mM Tris–HCl, 1% NP40, 150 mM NaCl, 10% glycerol, 1 mM Na_3_VO_4_, 50 mM NaF and Complete protease inhibitor (Roche Diagnostics, Mannheim, Germany). Lysates were denatured in 1× NuPage LDS sample buffer supplemented with 0.1 mM DTT for 10 min at 70°C before they were separated on 4-12% Bis‐Tris polyacrylamide gel. Proteins were transferred to a nitrocellulose membrane using the iBlot Dry Blotting System (Invitrogen, Camarillo, CA, USA). Membranes were blocked using 5% bovine serum albumin (Sigma–Aldrich, St. Louis, MO) in Tris‐buffered saline with 0.01% Tween followed by overnight incubation with the primary antibodies previously described. Detection was performed using horseradish peroxidase (HRP) conjugated antibodies (DAKO, Glostrup, Denmark) and developed with Super Signal West Femto Maximum Sensitivity Substrate (Thermo Scientific, Rockford, IL, USA). Images were obtained with LI‐COR Odyssey Fc and analyzed using Image Studio Software (LI‐COR, Lincoln, NE, USA).

### Statistical analyses

Results from *in vitro* experiments were graphed and analyzed using GraphPad by Prism version 8 software (La Jolla, CA, USA). Patient datasets were analyzed in R, with statistical packages. Experimental replicate numbers and statistical tests are indicated in the figure legends. Briefly, mean value and standard error of the mean (SEM) were calculated for replicates from independent experiments. For replicates within one experiment mean and SD is shown. For comparison of two groups unpaired Student´s t-test was used. For comparison of two groups with measurements over time, multiple t-tests were performed. For comparison of different treatments within one group one-way ANOVA and Dunnett´s multiple comparison was used. For comparison of more than two groups with measurements over time, two-way ANOVA and Dunnett´s multiple comparisons test was used.

## Results

### TLR-induced NFκB signaling promotes IL-32 expression in MM cells

**Figure 1:**
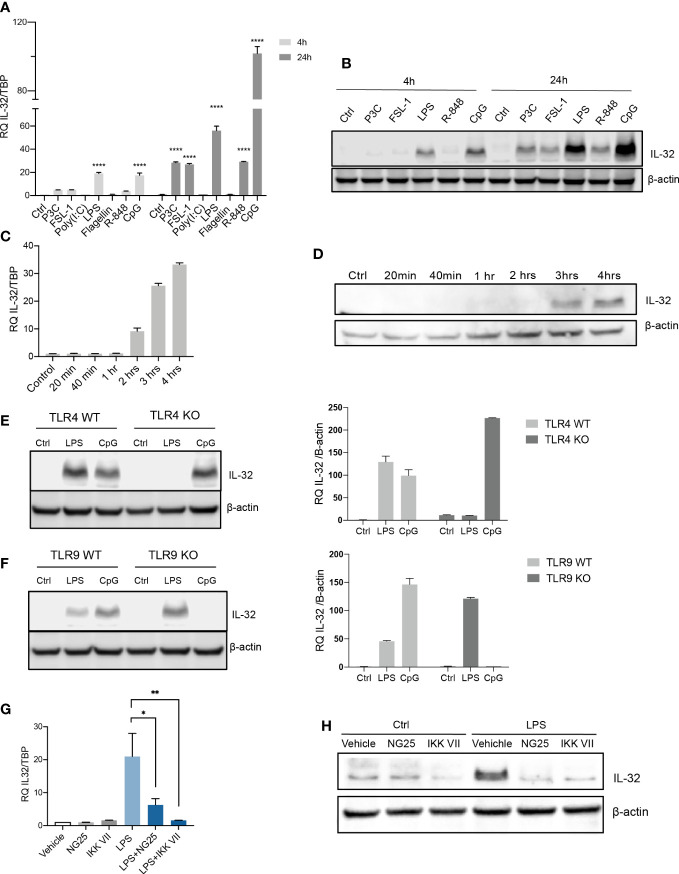
TLR-induced NFκB signaling promotes IL-32 expression in MM cells. **(A)** RPMI-8226 cells were stimulated with TLR agonists (for concentrations, see methods) for 4 and 24 hours and IL-32 mRNA expression was assessed by qPCR. The figure shows mean ± SEM of 3 independent experiments. **(B)** RPMI-8226 cells were stimulated with TLR agonists for 4 and 24 hours and IL-32 protein expression was evaluated by western blot. The figure shows representative western blot of 3 independent experiments. **(C)** RPMI-8226 cells were harvested at different time-points following LPS stimulation (0.1 µg/mL) and IL-32 mRNA expression was analyzed by qPCR (mean ± SD) and **(D)** IL-32 protein expression by western blot **(E)** RPMI-8226 TLR4 WT (mock) and KO cell lines were stimulated with LPS and CpG for 24 hours. Figure shows representative western blot (n=3) of IL-32 protein and qPCR analysis of IL-32 mRNA (mean ± SD, n=1) **(F)** RPMI-8226 TLR9 WT (mock) and KO cell lines were stimulated with LPS and CpG for 24 hours. The figure shows representative western blot (n=2) of IL-32 protein and qPCR analysis of IL-32 mRNA (mean ± SD, n=1) **(G)** RPMI-8226 cells were stimulated with LPS (0.1 µg/mL) and NG25 (2 µM) or IKK VII (10 µM) for 4 hours. IL-32 mRNA expression (mean ± SEM, n=3) was assessed by qPCR. **(H)** RPMI-8226 cells were stimulated with LPS, NG25 and IKK VII (concentrations as above) for 4 hours. The figure shows representative western blot (n=3) of IL-32 protein expression. P-values in **(A)** and **(G)** are calculated by one-way ANOVA with Dunnett´s multiple comparison test. *p≤ 0.05, **p ≤ 0.001, ****p ≤ 0.0001.

We previously showed that IL-32 is expressed in a subset of MM patients and MM cell lines (6). TLRs are also expressed by primary MM cells (21, 22). Both IL-32- and TLR expression varies between cell lines (**Supplemental Figures 1A, B**). RPMI-8226 cells have a broad repertoire of TLRs and do not express IL-32 in basal culture conditions (**Supplemental Figure 1**), and we therefore used these cells for TLR-stimulation. Optimal ligand concentrations used in the experiments were determined by titration (**Supplemental Figures 2A–C**). IL-32 mRNA was upregulated in RPMI-8226 in response to Pam3cys (TLR2/1), FSL-1 (TLR2/6), LPS (TLR4), R-848 (TLR7 and TLR8) and CpG (TLR9) (**Figure 1A**). Moreover, IL-32 protein expression was increased in response to LPS and CpG after 4 hours, and after 24 hours, increased IL-32 protein was evident also in response to Pam3cys, FSL-1 and R-848 (**Figure 1B**). Thus, increased IL-32 mRNA corresponded to increased IL-32 protein. IL-32 was transcribed after 2 hours of stimulation with LPS (**Figure 1C**), while IL-32 protein was evident after 3 hours, indicating that LPS-induced IL-32 is regulated through gene transcription (**Figure 1D**).

To investigate if IL-32 expression was induced by LPS and CpG through their cognate receptors, we evaluated the expression of IL-32 in RPMI-8226 cells depleted of TLR4 or TLR9 (validation of knock out in **Supplemental Figures 2D, E**). As expected, LPS did not promote IL-32 expression in TLR4 KO cells (**Figure 1E**), while the TLR9 ligand CpG did not induce IL-32 in TLR9 KO cells (**Figure 1F**), supporting that the induction of IL-32 in response to PAMPs is mediated by cognate TLR receptor-ligand interactions.

NFκB is a central downstream transcription factor of TLR-signaling. To investigate if transcription of IL-32 was regulated by NFκB we treated RPMI-8226 cells with two inhibitors of NFκB signaling: the IKK inhibitor VII and the TAK1/MAP4K2 inhibitor NG25. LPS-induced IL-32 was significantly reduced with NG25 and almost completely abolished by the IKK inhibitor VII, both at the mRNA level (**Figure 1G**; **Supplemental Figures 2F, G**)) and at the protein level (**Figure 1H**) indicating that NFκB is the main regulator of the LPS-induced transcription of IL-32. Expression of IL-32 following CpG stimulation was also inhibited by the IKK inhibitor, supporting that NFκB is essential for inducing IL-32 also in response to other TLR agonists (**Supplemental Figure 2H**). The NFκB inhibition was validated by western blotting of p-IκB where the LPS- induced increase of p-IκB was reduced upon treatment with IKK-VII (**Supplemental Figure 2I**). Induction of IL-32 by LPS, and inhibition of IL-32 expression by the NFκB inhibitor VII was also observed in PBMCs from healthy donors, supporting that the response may be conserved in different immune cells (**Supplemental Figures 2J, K**). Taken together, these results show that ligand binding and signal transduction through TLRs followed by activation of NFκB is important for the induction of IL-32 in MM cells in response to TLR agonists.

### IL-32 is associated with TLR expression in primary MM cells

**Figure 2:**
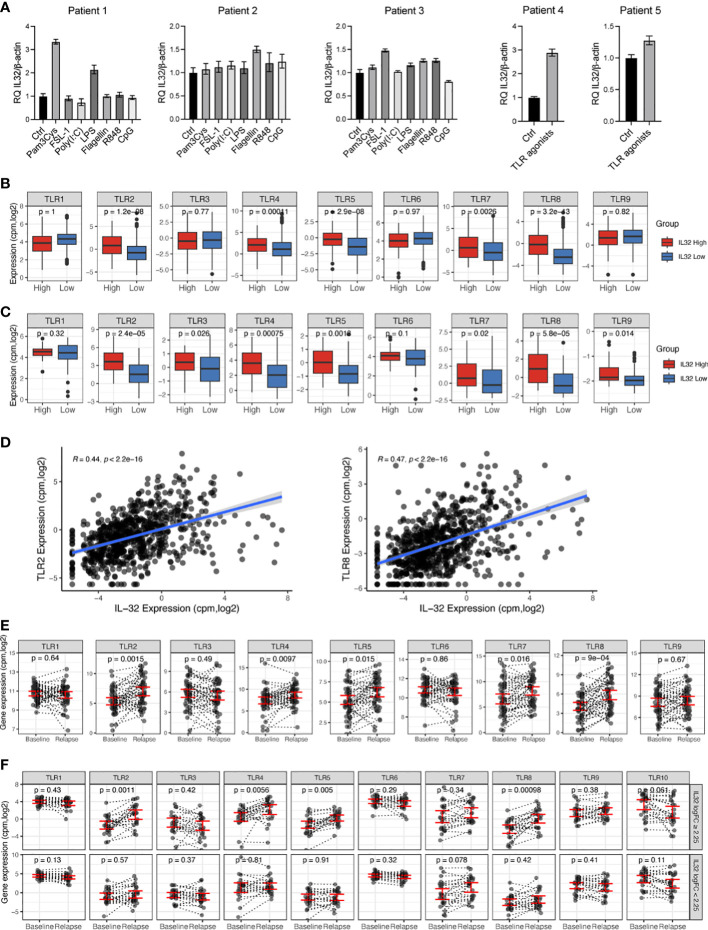
IL-32 is associated with TLR expression in primary MM cells. **(A)** Freshly obtained primary CD138+ myeloma cells were stimulated with TLR agonists for 4 hours and IL-32 expression was evaluated by qPCR. The figure shows mean RQ ± SD of technical replicates. The Ct-values for IL-32 in untreated cells were for patient #1: 33.13, patient #2: 34.05, patient #3: 31.32, patient # 4: 31.92 and patient #5: 32.43. **(B)** TLR gene expression in IL-32 expressing patients (upper10^th^ percentile) compared to non-expressing patients (lower 90^th^ percentile) in CoMMpass IA13. **(C)** TLR gene expression in IL-32 expressing patients (upper 30^th^ percentile) compared to non-expressing patients (lower 70^th^ percentile) in our own (Biobank1) dataset. **(D)** Plot showing Pearson correlation between IL-32 and TLR2 and TLR8 in the CoMMpass IA13 dataset. **(E)** TLR expression at diagnosis and first relapse timepoint in RNA-sequenced longitudinal CD138+ patient samples from CoMMpass IA13. Figure shows the mean ± SEM for TLR expression at diagnosis and relapse. **(F)** TLR expression between diagnosis (baseline) and relapse for patients with an FC ≥ 2.25 increase of IL-32 in relapse (n=24) and patients with FC<2.25 increase of IL-32 in relapse (n=23). The figure shows the mean ± SEM for TLR expression at diagnosis and relapse in each group. In A, B and D, significance was analyzed by one-tailed Wilcoxon signed-rank test. In E significance was calculated by two-tailed Wilcoxon signed-rank test.

As we found IL-32 to be upregulated by TLR activation *in vitro*, we next investigated if IL-32 is induced in primary myeloma cells in response to TLR agonists (**Figure 2A**). Freshly obtained CD138+ primary cells from five patients were stimulated with ligands activating TLR 1-9. IL-32 was increased more than two-fold in response to TLR1/2 agonist Pam3Cys and TLR4 agonist LPS in patient #1. In patients #2 and #3 Il-32 expression was increased about 50% in response to flagellin and FSL-1, respectively (**Figure 2A**). For patients #4 and #5, fewer cells were donated, and we therefore used a cocktail containing all agonists for stimulation. IL-32 expression increased nearly three-fold in patient #4 following TLR agonist cocktail stimulation, while the response to TLR stimulation was less prominent in patient #5 (about 30% percent increase in IL-32 mRNA expression).

To further assess the relationship between IL-32 and TLRs in myeloma patients we compared TLR gene expression in MM primary cells with high- (n=80) or no/low expression of IL-32 (n=712) (IA13 CoMMpass dataset, cutoff: log2cpm>1.52, upper 10^th^ percentile *vs*. lower 90^th^ percentile). We found that TLR2, TLR4, TLR5, TLR7 and TLR8 were significantly upregulated in IL-32-expressing plasma cells (**Figure 2B**). Upregulation of these TLRs in IL-32-expressing MM cells was confirmed in our own independent patient cohort (23) (**Figure 2C**). Of all TLR genes in the CoMMpass dataset, TLR2 (R=0.44, p= 2.2e-16) and TLR8 (R=0.47, p=2.2e-16) were the TLRs most highly correlated to IL-32 (**Figure 2D**). Thus, IL-32 expression is positively associated with TLR expression in patient samples.

We previously showed that IL-32 is upregulated upon relapse and that some IL-32-non-expressors start to express IL-32 in the time period between diagnosis and relapse (5). To investigate if TLR expression in a similar manner is increased in samples obtained at relapse compared with samples obtained at diagnosis we analyzed the same longitudinal RNA-sequenced samples (n=47). Indeed, TLR2, TLR4, TLR5, TLR7 and TLR8 mRNA expression were significantly upregulated at treatment relapse compared with expression at time of diagnosis (**Figure 2E**). Interestingly, these were the same TLRs that were most significantly associated with IL-32 in the diagnostic samples in the CoMMpass dataset (**Figure 2B**) as well as in our own dataset (**Figure 2C**). To investigate if the increased expression of TLRs in samples obtained at relapse coincided with the increase of IL-32, we divided the patient samples into two groups based on the fold change upregulation of IL-32 from diagnosis to relapse (logFC ≥ 2.25, n= 23 and log2FC<2.25, n=24, **Supplemental Table 1**) and compared changes in TLR expression between the two groups. Strikingly, patients with an IL-32 FC2.25 from diagnosis to relapse had significantly increased TLR 2-,4-,5- and 8- expression at relapse compared with expression at diagnosis. (**Figure 2F**). In contrast, in the group of patients with no/small increase in IL-32 in the relapse samples, there was no significant increase in TLR expression at relapse (**Figure 2E**). These data support that a subgroup of patients experience high IL-32 and high TLR expression upon disease progression.

Finally, as we found that NFκB transduced the TLR-dependent transcription of IL-32 we asked whether the fraction of patients with hyperactivated NFκB is also the IL-32 expressors. NFκB is constitutively active in 20% of MM patients and 40% of MM cell lines (24–26). We analyzed the IL-32 expression in patients from the CoMMpass IA11 dataset with and without a mutation in the NFκB pathway (27). There was however no association between IL-32 expression and mutated NFκB (**Supplemental Figure 2L**), indicating that hyperactivated NFκB is not the leading cause of constitutive IL-32 expression in MM patients.

## Discussion

Here we demonstrate that that IL-32 is induced in MM cells in response to PAMPs through NF-κB activation downstream of TLRs. We further found that IL-32 expression is associated with TLR expression in MM patients in two large, independent patient cohorts. We also show that TLR expression in malignant plasma cells increased from diagnosis to relapse in individual patients and coincided with an increase in IL-32.

IL-32 is expressed in 10-20% of patients at diagnosis, and a larger fraction of patients start to express IL-32 following treatment (5). Importantly, the expression is not linked to specific genetic subgroups (6), which supports that IL-32 expression is induced by extracellular cues. Indeed, IL-32 is upregulated in MM cells by hypoxia (6), which is a characteristic of the MM bone marrow (6, 28–30). Our *in vitro* and *in vivo* data presented here support that IL-32 expression in MM cells can also be induced by TLR-activation. TLRs are heterogeneously expressed by primary MM cells (21, 22, 31) and exposure of various TLR-ligands is likely to have different effects depending on the cells’ TLR repertoire (32). Indeed, this is evident in our experiments with primary cells, where cells from all patients increased IL-32 mRNA in response to TLR-stimulation, but which ligands that activated the cells and to which extent IL-32 was increased varied greatly. In patient sequencing data the strongest correlations were between IL-32 and TLR2 and TLR8.

In the bone marrow, TLR agonists may be derived from pathogens or from damaged or dead cells. TLR expression has been shown to increase upon infection (33–35). Thus, although this correlation is not absolute (36), high expression of TLRs in primary MM cells may be indicative of an inflammatory bone marrow microenvironment or of an ongoing infection. MM patients are susceptible to infections (16) and the anti-MM treatments increase the frequency of infection further (37). The level of DAMPs rises following anti-MM treatment due to tumor cell death. It is therefore likely that DAMPs may also be a driver of IL-32 expression in the MM bone marrow microenvironment, but this remains to be further investigated.

Intracellular IL-32 promotes proliferation and survival of MM cells (5, 38) and depletion of IL-32 from myeloma cell lines reduced tumor engraftment and/or tumor growth in three different xenograft mouse models(5). At the molecular level, IL-32 interacts with components of the mitochondrial respiratory chain and promotes oxidative phosphorylation in malignant plasma cells (5). IL-32 is also expressed in plasma cells obtained from healthy individuals (5). However, whether IL-32 is induced to a similar extent upon TLR activation in non-malignant plasma cells and the role of IL-32 for normal plasma cell function needs to be elucidated.

In the cancer setting, MM cell-derived IL-32 may also have indirect effects on tumor progression. IL-32 can accelerate disease progression by promoting osteoclast differentiation and bone destruction (6.) In an early study, IL-32 was shown to promote the differentiation of monocytes into macrophages while the generation of functional dendritic cells was inhibited (39), which may suggest that IL-32 can have negative effects on immune responses. Indeed, more recently, myeloma-derived IL-32 was shown to promote the formation of immunosuppressive, M2-like macrophages (8, 40, 41).

In conclusion, activation of TLRs in an inflamed or infectious bone marrow microenvironment may lead to IL-32 expression, and this may contribute to accelerate the disease. We therefore propose that the subgroup of IL-32-expressing patients may benefit from combination treatments where drugs targeting hypoxia, antibiotics, antiviral- or anti-inflammatory drugs are included.

## Data availability statement

The original contributions presented in the study are publicly available. This data can be found here: https://github.com/MjelleLab/MicroRNA-and-Gene-Expression-In-Multiple-Myeloma/blob/master/counts_standal_biobank_ready.cpm.merge.cpgz and https://portal.gdc.cancer.gov/projects/MMRF-COMMPASS.

## Ethics statement

The studies involving human participants were reviewed and approved by the Regional Ethics Committee 247909. The patients/participants provided their written informed consent to participate in this study.

## Author contributions

KA and ST designed experiments; KA, ST, RM, and TN conducted experiments, acquired, and analyzed data; KS organized the longitudinal CoMMpass data; TS designed the study and received funding; KA and TS wrote the manuscript. All authors contributed to the article and approved the submitted version.

## References

[1] Rajkumar SV. Multiple myeloma: 2020 update on diagnosis, risk-stratification and management. Am J Hematol (2020) 95:548–67. doi: 10.1002/ajh.25791 32212178

[2] Kim SH Han SY Azam T Yoon DY Dinarello CA. Interleukin-32: a cytokine and inducer of TNFalpha. Immunity (2005) 22:131–42. doi: 10.1016/j.immuni.2004.12.003 15664165

[3] Ribeiro-Dias F Saar Gomes R De Lima Silva LL Dos Santos JC Joosten LA. Interleukin 32: A novel player in the control of infectious diseases. J Leukoc Biol (2017) 101:39–52. doi: 10.1189/jlb.4RU0416-175RR 27793959

[4] Aass KR Kastnes MH Standal T. Molecular interactions and functions of IL-32. J Leukocyte Biol (2021) 109:143–59. doi: 10.1002/JLB.3MR0620-550R 32869391

[5] Aass KR Mjelle R Kastnes MH Tryggestad SS Van Den Brink LM Roseth IA. Intracellular IL-32 regulates mitochondrial metabolism, proliferation and differentiation of malignant plasma cells. iScience (2021) 25(1):103605. doi: 10.1016/j.isci.2021.103605 35005550 PMC8717606

[6] Zahoor M Westhrin M Aass KR Moen SH Misund K Psonka-Antonczyk KM. Hypoxia promotes IL-32 expression in myeloma cells, and high expression is associated with poor survival and bone loss. Blood Adv (2017) 1:2656–66. doi: 10.1182/bloodadvances.2017010801 PMC5745138 29296919

[7] Yan H Dong M Liu X Shen Q He D Huang X. Multiple myeloma cell-derived IL-32γ increases the immunosuppressive function of macrophages by promoting indoleamine 2,3-dioxygenase (IDO) expression. Cancer Lett (2019) 446:38–48. doi: 10.1016/j.canlet.2019.01.012 30660652

[8] Liu Y Yan H Gu H Zhang E He J Cao W. Myeloma-derived IL-32γ induced PD-L1 expression in macrophages facilitates immune escape *via* the PFKFB3-JAK1 axis. Oncoimmunology (2022) 11:2057837. doi: 10.1080/2162402X.2022.2057837 35371618 PMC8973380

[9] Li W Liu Y Mukhtar MM Gong R Pan Y Rasool ST. Activation of interleukin-32 pro-inflammatory pathway in response to influenza a virus infection. PLoS One (2008) 3:e1985. doi: 10.1371/journal.pone.0001985 18414668 PMC2288676

[10] Schenk M Krutzik SR Sieling PA Lee DJ Teles RM Ochoa MT. NOD2 triggers an interleukin-32-dependent human dendritic cell program in leprosy. Nat Med (2012) 18:555–63. doi: 10.1038/nm.2650 PMC3348859 22447076

[11] Zhang L Che C Lin J Liu K Li DQ Zhao G. TLR-mediated induction of proinflammatory cytokine IL-32 in corneal epithelium. Curr Eye Res (2013) 38:630–8. doi: 10.3109/02713683.2012.763102 23534905

[12] Wang X Sjölinder M Gao Y Wan Y Sjölinder H. Immune homeostatic macrophages programmed by the bacterial surface protein NhhA potentiate nasopharyngeal carriage of &lt;span class=&quot;named-content genus-species&quot; id=&quot;named-content-1&quot;<Neisseria meningitidis&lt;/span&gt. mBio (2016) 7:e01670–01615. doi: 10.1128/mBio.01670-15 PMC4752598 26884432

[13] Silveira MB Gomes RS Shio MT Rugani JN Paranaiba LF Soares RP. Lipophosphoglycan from dermotropic new world leishmania upregulates interleukin-32 and proinflammatory cytokines through TLR4 and NOD2 receptors. Front Cell Infection Microbiol (2022) 12. doi: 10.3389/fcimb.2022.805720 PMC8983857 35402314

[14] Piccinini AM Midwood KS. DAMPening inflammation by modulating TLR signalling. Mediators Inflammation (2010) 2010:672395. doi: 10.1155/2010/672395 PMC2913853 20706656

[15] Jang G-Y Lee JW Kim YS Lee SE Han HD Hong K-J. Interactions between tumor-derived proteins and toll-like receptors. Exp Mol Med (2020) 52:1926–35. doi: 10.1038/s12276-020-00540-4 PMC8080774 33299138

[16] Blimark C Holmberg E Mellqvist UH Landgren O Björkholm M Hultcrantz M. Multiple myeloma and infections: a population-based study on 9253 multiple myeloma patients. Haematologica (2015) 100:107–13. doi: 10.3324/haematol.2014.107714 PMC4281323 25344526

[17] Teh BW Harrison SJ Slavin MA Worth LJ. Epidemiology of bloodstream infections in patients with myeloma receiving current era therapy. Eur J Haematol (2017) 98:149–53. doi: 10.1111/ejh.12813 27717026

[18] Lin C Shen H Zhou S Liu M Xu A Huang S. Assessment of infection in newly diagnosed multiple myeloma patients: risk factors and main characteristics. BMC Infect Dis (2020) 20:699. doi: 10.1186/s12879-020-05412-w 32972385 PMC7517606

[19] Hjertner OY Hjorth-Hansen H Börset M Seidel C Waage A Sundan A. Bone morphogenetic protein-4 inhibits proliferation and induces apoptosis of multiple myeloma cells: Presented in part at the 41st annual meeting of the American society of hematology, new Orleans, December 1999. Blood (2001) 97:516–22. doi: 10.1182/blood.V97.2.516 11154231

[20] Vatsveen TK Tian E Kresse SH Meza-Zepeda LA Gabrea A Glebov O. OH-2, a hyperdiploid myeloma cell line without an IGH translocation, has a complex translocation juxtaposing MYC near MAFB and the IGK locus. Leuk Res (2009) 33:1670–7. doi: 10.1016/j.leukres.2009.03.001 PMC6944273 19395026

[21] Bohnhorst J Rasmussen T Moen SH Flottum M Knudsen L Borset M. Toll-like receptors mediate proliferation and survival of multiple myeloma cells. Leukemia (2006) 20:1138–44. doi: 10.1038/sj.leu.2404225 16617319

[22] Bagratuni T Sklirou AD Kastritis E Liacos CI Spilioti C Eleutherakis-Papaiakovou E. Toll-like receptor 4 activation promotes multiple myeloma cell growth and survival *Via* suppression of the endoplasmic reticulum stress factor chop. Sci Rep (2019) 9:3245. doi: 10.1038/s41598-019-39672-7 30824741 PMC6397208

[23] Aass KR Nedal TMV Tryggestad SS Haukås E Slørdahl TS Waage A. Paired miRNA- and messenger RNA-sequencing identifies novel miRNA-mRNA interactions in multiple myeloma. Sci Rep (2022) 12:12147. doi: 10.1038/s41598-022-16448-0 35840794 PMC9287335

[24] Annunziata CM Davis RE Demchenko Y Bellamy W Gabrea A Zhan F. Frequent engagement of the classical and alternative NF-kappaB pathways by diverse genetic abnormalities in multiple myeloma. Cancer Cell (2007) 12:115–30. doi: 10.1016/j.ccr.2007.07.004 PMC2730509 17692804

[25] Keats JJ Fonseca R Chesi M Schop R Baker A Chng W-J. Promiscuous mutations activate the noncanonical NF-kappaB pathway in multiple myeloma. Cancer Cell (2007) 12:131–44. doi: 10.1016/j.ccr.2007.07.003 PMC2083698 17692805

[26] Demchenko YN Glebov OK Zingone A Keats JJ Bergsagel PL Kuehl WM. Classical and/or alternative NF-kappaB pathway activation in multiple myeloma. Blood (2010) 115:3541–52. doi: 10.1182/blood-2009-09-243535 PMC2867265 20053756

[27] Misund K Keane N Stein CK Asmann YW Day G Welsh S. MYC dysregulation in the progression of multiple myeloma. Leukemia (2020) 34:322–6. doi: 10.1038/s41375-019-0543-4 PMC6923575 31439946

[28] Colla S Storti P Donofrio G Todoerti K Bolzoni M Lazzaretti M. Low bone marrow oxygen tension and hypoxia-inducible factor-1α overexpression characterize patients with multiple myeloma: role on the transcriptional and proangiogenic profiles of CD138+ cells. Leukemia (2010) 24:1967. doi: 10.1038/leu.2010.193 20811474

[29] Azab AK Hu J Quang P Azab F Pitsillides C Awwad R. Hypoxia promotes dissemination of multiple myeloma through acquisition of epithelial to mesenchymal transition-like features. Blood (2012) 119:5782–94. doi: 10.1182/blood-2011-09-380410 PMC3382938 22394600

[30] Maiso P Huynh D Moschetta M Sacco A Aljawai Y Mishima Y. Metabolic signature identifies novel targets for drug resistance in multiple myeloma. Cancer Res (2015) 75:2071–82. doi: 10.1158/0008-5472.CAN-14-3400 PMC4433568 25769724

[31] Xu Y Zhao Y Huang H Chen G Wu X Wang Y. Expression and function of toll-like receptors in multiple myeloma patients: toll-like receptor ligands promote multiple myeloma cell growth and survival *via* activation of nuclear factor-κB. Br J Haematology (2010) 150:543–53. doi: 10.1111/j.1365-2141.2010.08284.x 20629663

[32] Akesolo O Buey B Beltrán-Visiedo M Giraldos D Marzo I Latorre E. Toll-like receptors: New targets for multiple myeloma treatment? Biochem Pharmacol (2022) 199:114992. doi: 10.1016/j.bcp.2022.114992 35292256

[33] Miettinen M Sareneva T Julkunen I Matikainen S. IFNs activate toll-like receptor gene expression in viral infections. Genes Immun (2001) 2:349–55. doi: 10.1038/sj.gene.6363791 11607792

[34] Zarember KA Godowski PJ. Tissue expression of human toll-like receptors and differential regulation of toll-like receptor mRNAs in leukocytes in response to microbes, their products, and cytokines. J Immunol (2002) 168:554. doi: 10.4049/jimmunol.168.2.554 11777946

[35] Hadley JS Wang JE Foster SJ Thiemermann C Hinds CJ. Peptidoglycan of staphylococcus aureus upregulates monocyte expression of CD14, toll-like receptor 2 (TLR2), and TLR4 in human blood: Possible implications for priming of lipopolysaccharide signaling. Infect Immun (2005) 73:7613–9. doi: 10.1128/IAI.73.11.7613-7619.2005 PMC1273841 16239565

[36] Anwar MA Basith S Choi S. Negative regulatory approaches to the attenuation of toll-like receptor signaling. Exp Mol Med (2013) 45:e11–1. doi: 10.1038/emm.2013.28 PMC3584666 23429360

[37] Nucci M Anaissie E. Infections in patients with multiple myeloma in the era of high-dose therapy and novel agents. Clin Infect Dis (2009) 49:1211–25. doi: 10.1086/605664 19769539

[38] Lin X Yang L Wang G Zi F Yan H Guo X. Interleukin-32α promotes the proliferation of multiple myeloma cells by inducing production of IL-6 in bone marrow stromal cells. Oncotarget (2017) 8:92841–54. doi: 10.18632/oncotarget.21611 PMC5696226 29190960

[39] Netea MG Lewis EC Azam T Joosten LA Jaekal J Bae SY. Interleukin-32 induces the differentiation of monocytes into macrophage-like cells. Proc Natl Acad Sci U.S.A. (2008) 105:3515–20. doi: 10.1073/pnas.0712381105 PMC2265135 18296636

[40] Yan H Dong M Liu X Shen Q He D Huang X. Multiple myeloma cell-derived IL-32gamma increases the immunosuppressive function of macrophages by promoting indoleamine 2,3-dioxygenase (IDO) expression. Cancer Lett (2019) 446:38–48. doi: 10.1016/j.canlet.2019.01.012 30660652

[41] Yan H He D Qu J Liu Y Xu R Gu H. Interleukin-32γ promotes macrophage-mediated chemoresistance by inducing CSF1-dependent M2 macrophage polarization in multiple myeloma. Cancer Immunol Immunother (2023) 72:327–38. doi: 10.1007/s00262-022-03241-1 PMC10991222 35881196

