# Supplementary figures and images for: IL-32 is induced by activation of toll-like receptors in multiple myeloma cells
Source: Front Immunol. 2023 Feb 16;14:1107844. doi: 10.3389/fimmu.2023.1107844 (PMC9978100; doi:10.3389/fimmu.2023.1107844)

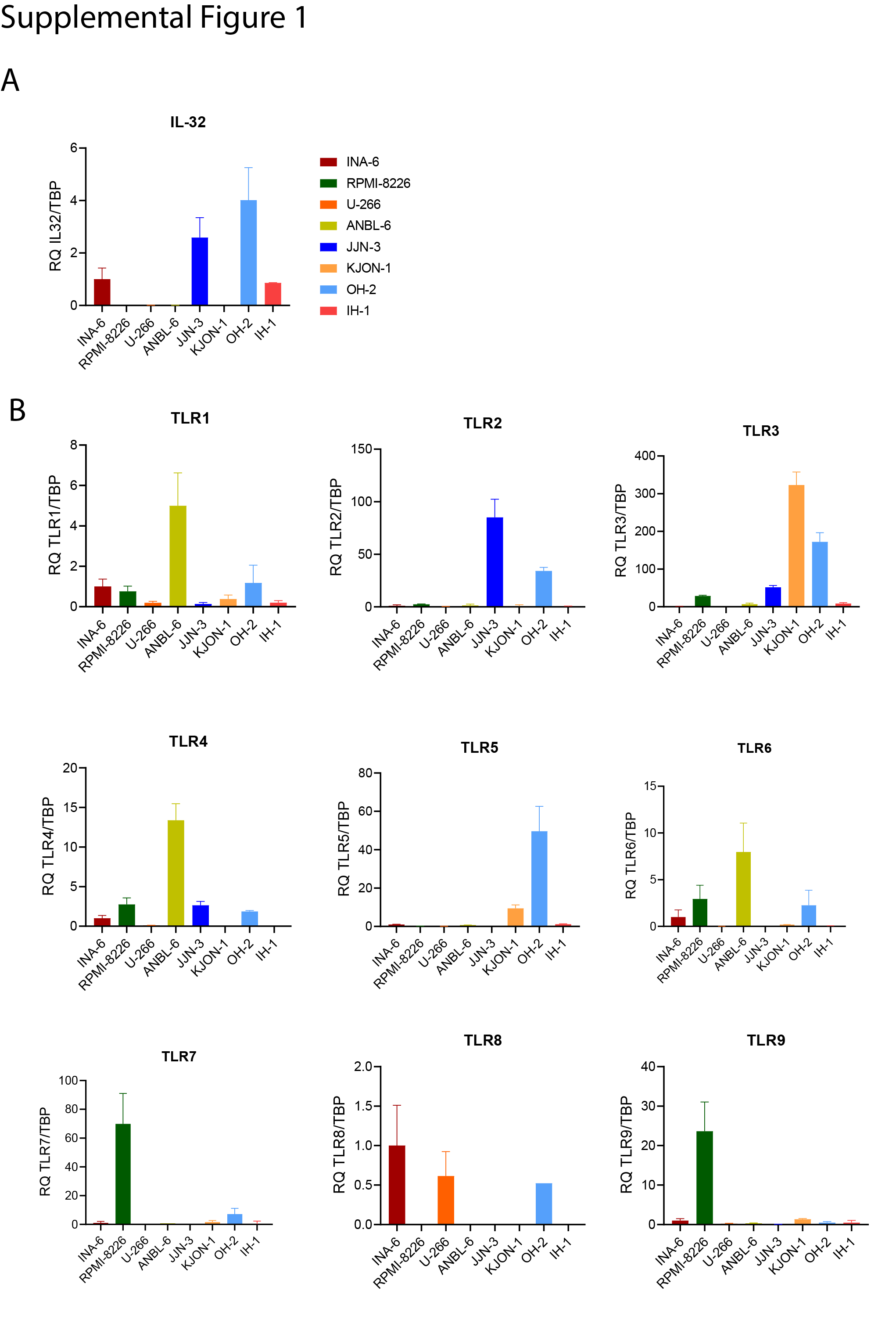

Supplement: Supplementary file 2 [file Image_1.tif]

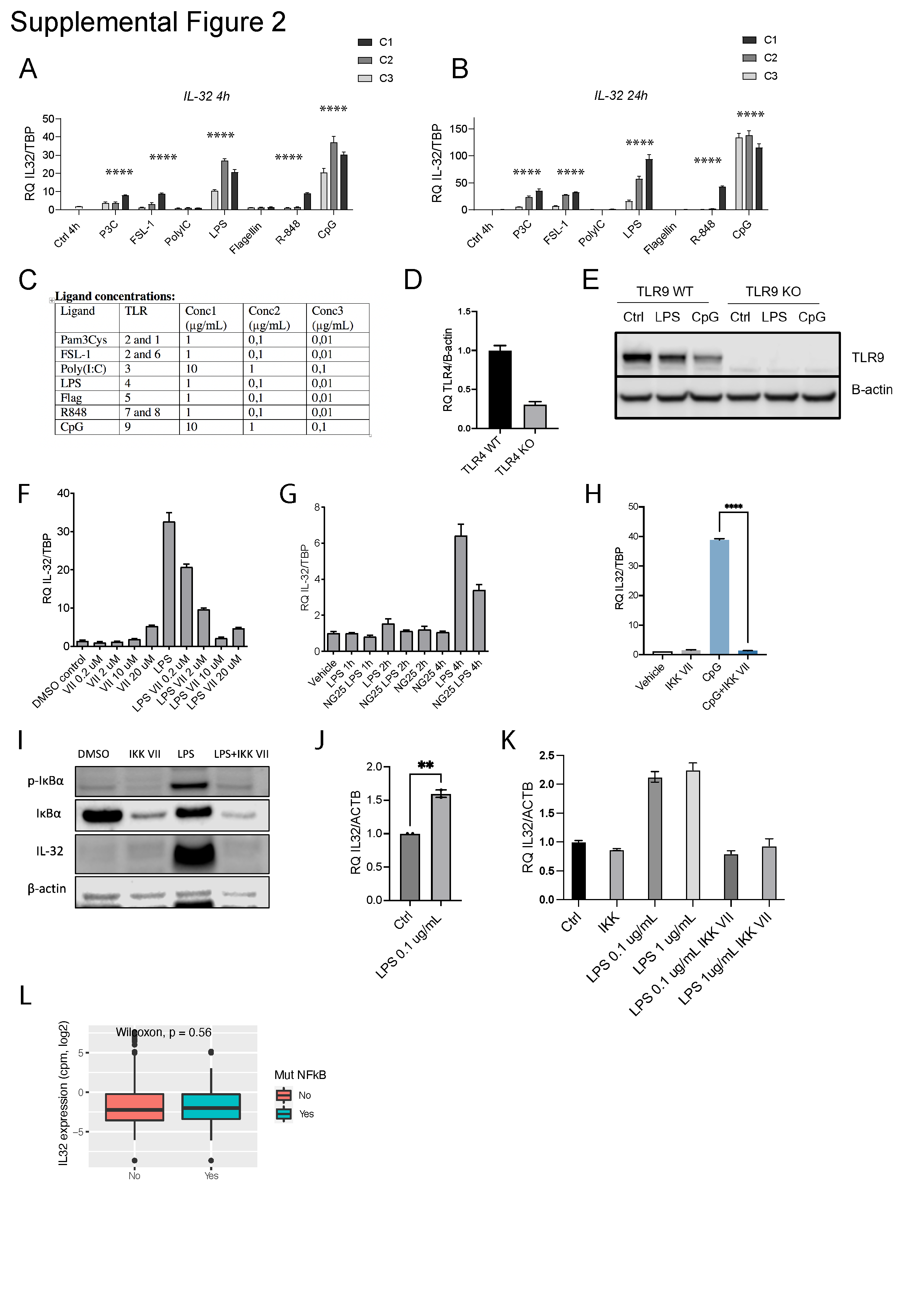

Supplement: Supplementary file 3 [file Image_2.tiff]
